# Synthesis and characterizations of Ni-NiO nanoparticles on PDDA-modified graphene for oxygen reduction reaction

**DOI:** 10.1186/1556-276X-9-444

**Published:** 2014-08-28

**Authors:** Tung-Yuan Yung, Li-Ying Huang, Tzu-Yi Chan, Kuan-Syun Wang, Ting-Yu Liu, Po-Tuan Chen, Chi-Yang Chao, Ling-Kang Liu

**Affiliations:** 1 Department of Physics, National Central University, Jhongli, Taoyuan 320, Taiwan; 2 Molecular Science and Technology, Taiwan International Graduate Program/Institute of Chemistry, Academia Sinica, Taipei 115, Taiwan; 3 Nuclear Fuels and Materials Division, Institute of Nuclear Energy Research, Lontan, Taoyuan 325, Taiwan; 4 Department of Materials Science and Engineering, National Taiwan University of Science and Technology, Taipei 106, Taiwan; 5 Department of Materials Engineering, Ming Chi University of Technology, New Taipei City 24301, Taiwan; 6 Center for Condensed Mater Sciences, National Taiwan University, Taipei 106, Taiwan; 7 Materials Science and Engineering, National Taiwan University, Taipei 106, Taiwan

**Keywords:** Graphene, PDDA, Ni, NiO, ORR, Fuel cells

## Abstract

We are presenting our recent research results about the Ni-NiO nanoparticles on poly-(diallyldimethylammonium chloride)-modified graphene sheet (Ni-NiO/PDDA-G) nanocomposites prepared by the hydrothermal method at 90°C for 24 h. The Ni-NiO nanoparticles on PDDA-modified graphene sheets are measured by transmission electron microscopy (TEM), energy-dispersive X-ray spectroscopy (EDS), and selected area electron diffraction (SAED) pattern for exploring the structural evidence to apply in the electrochemical catalysts. The size of Ni-NiO nanoparticles is around 5 nm based on TEM observations. The X-ray diffraction (XRD) results show the Ni in the (012), (110), (110), (200), and (220) crystalline orientations, respectively. Moreover, the crystalline peaks of NiO are found in (111) and (220). The thermal gravimetric analysis (TGA) result represents the loading content of the Ni metal which is about 34.82 wt%. The electron spectroscopy for chemical analysis/X-ray photoelectron spectroscopy (ESCA/XPS) reveals the Ni^0^ to Ni^II^ ratio in metal phase. The electrochemical studies with Ni-NiO/PDDA-G in 0.5 M aqueous H_2_SO_4_ were studied for oxygen reduction reaction (ORR).

## Background

Although platinum-based nanoparticles are thought as the best catalysts for oxygen reduction reaction (ORR), the increasing cost of Pt and the low abundance triggered scientists to develop non-noble metal catalysts for fuel cell applications [1-4]. The transition metal-based catalysts (based on Co, Ni, and Fe) are considered as a promising alternative due to their cheap cost and availability and have thus been studied for decades [5,6].

Catalysts for ORR of fuel cells (PEMFC and DMFC) have been the focus in recent years from the combination of Pt with varying metals to non-Pt-based metals [7-9]. Furthermore, carbon-supported nanocatalysts are also of great interest for scientists and engineers [7,10-14]. The ORR cathode is 6 or more orders of magnitude slower than the anode hydrogen oxidation reaction and thus limits performance, so almost all research and development focus on improving the cathode catalysts and electrodes [5]. The ORR catalysts are considered for mass production with the following factors: lower production of H_2_O_2_ during the ORR and higher tolerance of the impurities (Cl^−^ for instance). They must have the satisfied durability, and must be cost-effective. The three phenomena which lower the performance of fuel cells are kinetic losses, mass transport losses, and iR losses [5,7,15,16]. The ORR dominates the kinetic loss of fuel cells because the enhancement of the ORR activity would gain only 60 to 70 mV and kinetic losses are challenging. Moreover, the progress in catalyst development so far has achieved only modest cell voltage gains of tens of millivolts [5,17-19]. How to improve and enhance the catalyst electrochemical performances is the focus of scientists and engineers. Carbon-supported materials were introduced for fuel cell application. The supported materials would provide the surfaces for anchoring the catalysts and increasing the surface areas of the catalysts. Also, the supported material provides higher volume-to-mass ratio to make a good dispersive paste for electrode assembly. The size of Pt nanoparticles for the commercial Pt on carbon (Pt/C) is about 2 to 5 nm [5,20]. In addition to that, the Pt-based bimetallic system is interesting for ORR application, and the Pt_3_Ni bimetallic electrocatalyst on carbon support has also been known to serve as a catalyst for ORR [21]. Herein, we introduced additionally poly-(diallyldimethylammonium chloride) (PDDA) which further assists in the formation of a layer-to-layer structure for graphene surface modification (PDDA-G) on carbon-supported materials [22-25]. The synthesis of Ni-NiO nanoparticles on PDDA-G is done using the hydrothermal method. The results on hydrothermal synthesis of the Ni-NiO nanoparticles on PDDA-modified graphene for ORR application would be presented in this study.

## Methods

Graphene was prepared from graphite using the microwave synthesis method. Graphite (0.1 g; Sigma-Aldrich Co., St. Louis, MO, USA) was put into a 25-mL round-bottomed flask, and the flask was treated using a CEM Discover Du7046 microwave synthesis system (CEM, Matthews, NC, USA) with a power output of 20 W at 80°C for 10 s. The graphene was produced in 2 to 5 s with a sound of a bomb. Fifty milliliters of 3.5 wt% aqueous PDDA (Sigma-Aldrich) and 100 mg of graphene prepared by the method as mentioned were put into a 100-mL flask and then heated at 90°C for 4 h with a flux apparatus.

About 0.45 mmol Ni(NO_3_)_2_ · 2.5H_2_O was added into the above mentioned PDDA-G solution, followed by the addition of hydrazine hydrate of about 20 mmol. Then, the mixed solution was transferred into a Teflon-lined autoclave and heated at 90°C for 24 h. The mixture was centrifuged and washed for three times prior to drying at 90°C to produce the Ni-NiO nanoparticles on the PDDA-modified graphene (Ni-NiO/PDDA-G).

The crystalline structure of Ni-NiO/PDDA-G was examined by X-ray diffraction (XRD) using a Bruker D8 diffractometer (Bruker AXS, Karlsruhe, Germany) equipped with CuKα X-ray source. The chemical environments of Ni-NiO/PDDA-G were analyzed by electron spectroscopy for chemical analysis/X-ray photoelectron spectroscopy (ESCA/XPS) using a Thermo VG ESCAlab 250 (Thermo Fisher Scientific, Waltham, MA, USA) equipped with a dual-anode (MgKα/AlKα) X-ray source.

The microstructures of Ni-NiO/PDDA-G were investigated with the high-resolution microstructural images produced using the JOEL FEM 2100F (JEOL Ltd., Akishima, Tokyo, Japan) equipped with an Oxford energy-dispersive X-ray spectroscope (EDS) for element analysis.

Thermal gravimetric analysis (TGA) for nanoparticle loading was carried out using a PerkinElmer Pyris 1 instrument (PerkinElmer, Waltham, MA, USA) and by applying a heating rate of 10°C/min from room temperature to 800°C in an oxygen-purged environment.

The ORR study was examined using an Autolab potentiostat/galvanostat PGSTAT30 (Eco Chemie BV, Utrecht, The Netherlands). The reference electrode is Ag/AgCl (ALS Co. Ltd., Tokyo, Japan), and the counter electrode is a 0.5 mm × 10 cm platinum wire. The working electrode is the glassy carbon whose surface is deposited 5.24 μg/cm^2^ of Ni-NiO/PDDA-G. Cyclic voltammetry was used to investigate the 0.5 M aqueous H_2_SO_4_ and O_2_-saturated 0.5 M aqueous H_2_SO_4_ with a scanning rate of 50 mV/s. The electrochemical impedance spectroscopy (EIS) is also used as a test with an amplitude of 10 mV from 1 to 100 mHz.

## Results and discussion

**Figure 1:**
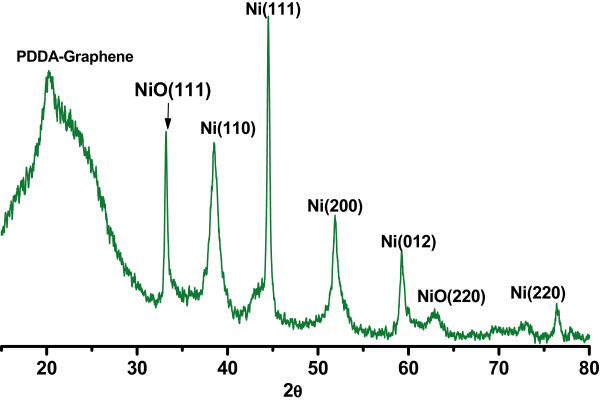
XRD patterns of Ni-NiO/PDDA-G nanohybrids.

**Figure 2:**
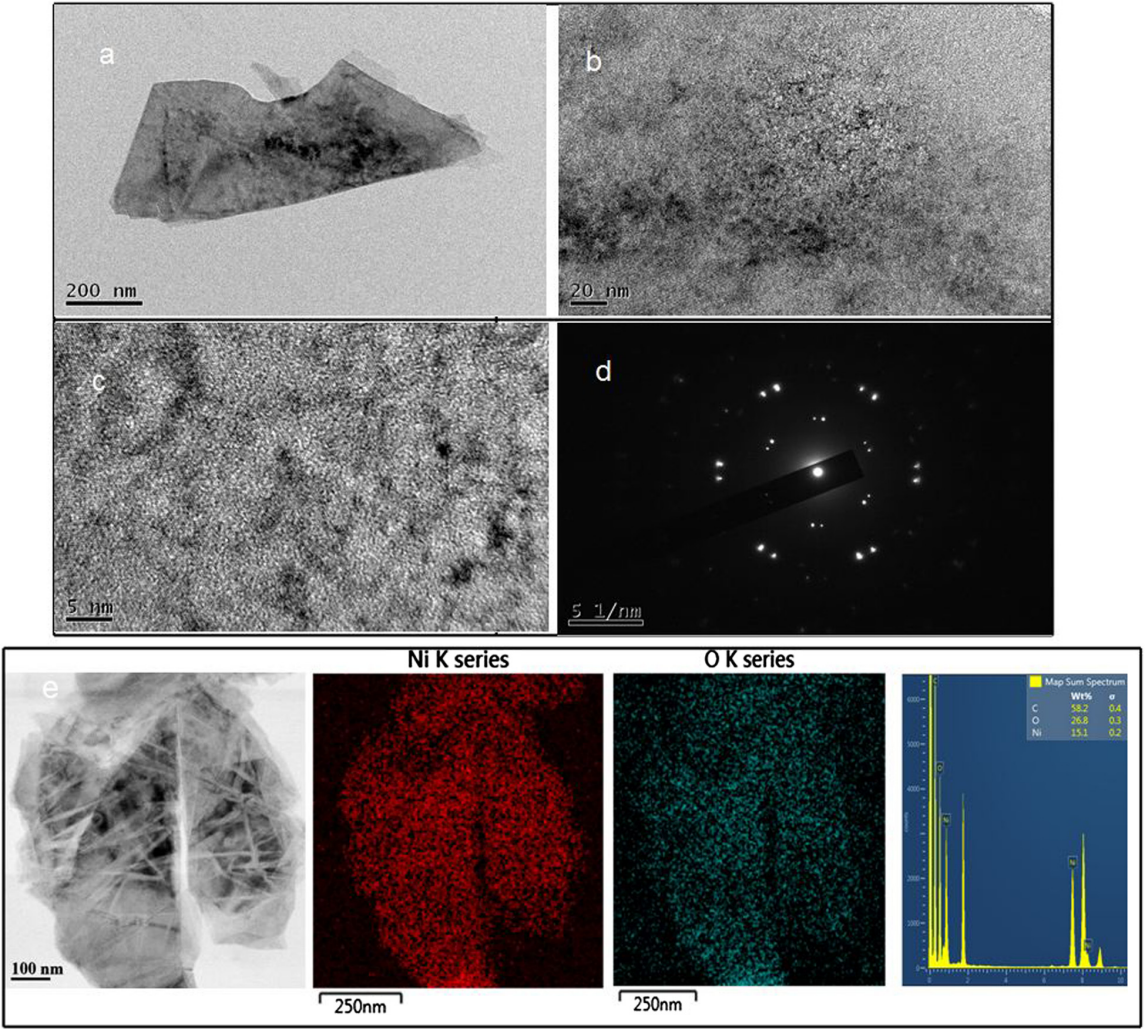
**TEM images and SAED pattern of Ni-NiO/PDDA-G nanohybrids. (a)** The low-magnification image of Ni-NiO/PDDA-G. **(b)** The high-magnification image of Ni-NiO/PDDA-G. **(c)** The high-resolution image of Ni-NiO/PDDA-G. **(d)** The SAED pattern of Ni-NiO/PDDA-G. **(e)** From left to right: STEM image, Ni element EDS mapping, O element EDS mapping, and the EDS spectrum of STEM-EDS mapping for Ni-NiO/PDDA-G, respectively.

**Figure 3:**
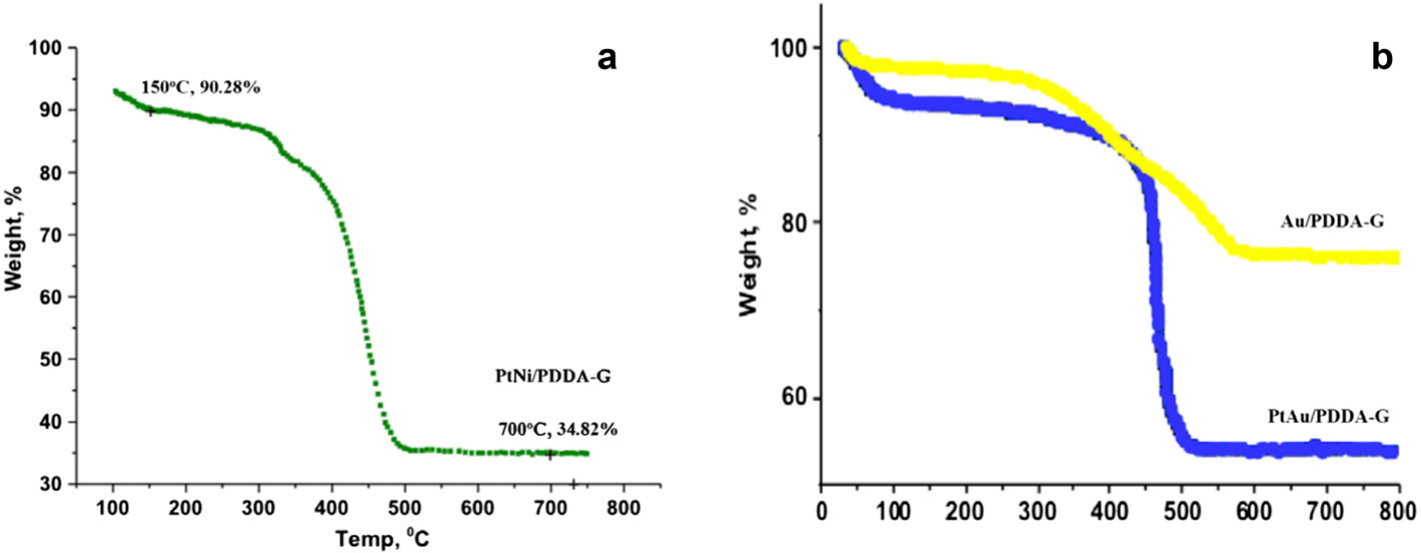
**TGA result of Ni-NiO/PDDA-G nanohybrids. (a)** Ni-NiO/PDDA-G. **(b)** The PtAu/PDDA-G and Au/PDDA-G.

The crystallization of Ni-NiO/PDDA-G was examined by XRD as shown in Figure 1. The peaks of the (002) plane in the PDDA-modified graphene was shifted from 20.5° to 22°, which revealed the change in the layer-to-layer distance of graphene due to incorporation of PDDA [21]. The hydrothermal method for synthesis of the Ni-NiO alloy nanoparticles was one-pot synthesis with a mixture of PDDA-G, Ni precursors, and hydrazine hydrates at 90°C for 24 h. The XRD result of Ni-NiO/PDDA-G indicated peaks assigned as Ni (111), Ni (200), Ni (012), Ni (222), NiO (111), NiO (012) and NiO (220), respectively [26,27].The microstructures of the Ni-NiO/PDDA-G nanohybrids are shown in Figure 2. The PDDA-modified graphene is a layer-by-layer structure, shown in Figure 2a. The Ni-NiO nanoparticles are anchoring between the layers and the surfaces of PDDA-G. Figure 2b,c shows the high-resolution TEM images for Ni-NiO/PDDA-G. The different contrasts are shown: Ni (dark) and NiO (bright) nanoparticles. Both particle sizes are around 2 to 5 nm. Selected area electron diffraction (SAED) patterns for the Ni and NiO are shown in Figure 2d. The brighter and bigger spots are for the Ni nanoparticle electron diffraction patterns. The results of EDS mapping from the STEM method are shown in Figure 2e. The Ni and O elements are colored red and blue to show the contribution for Ni-NiO nanoparticles on PDDA-G. The more condensed Ni element mapping is showing that the Ni-NiO nanoparticles exist. By EDS, the semi-quantified element ratios are Ni 15.1% and O 26.8% by weight (Ni 3.83% and O 24.7% by mole). The one-step synthesis with hydrothermal method is perfect for the synthesis process for the narrow size distribution of nanoparticles.TGA shows that the loading content of the Ni-NiO nanoparticles is about 34.84 wt% on the PDDA-G surfaces. The TGA result is shown in the Figure 3a. For comparison with the other metal loading contents by hydrothermal method, the Au/PDDA-G and PtAu/PDDA-G are observed in the Figure 3b. The same precursor loading (approximately 0.456 mmol) with the same batch PDDA-G was applied in the one-pot synthesis method. The nickel reduction rate is obviously lower than the reduction rate of gold and platinum by the metal loading amounts, which is in the order of 34.82, 58.2, and 74.1 wt%.

**Figure 4:**
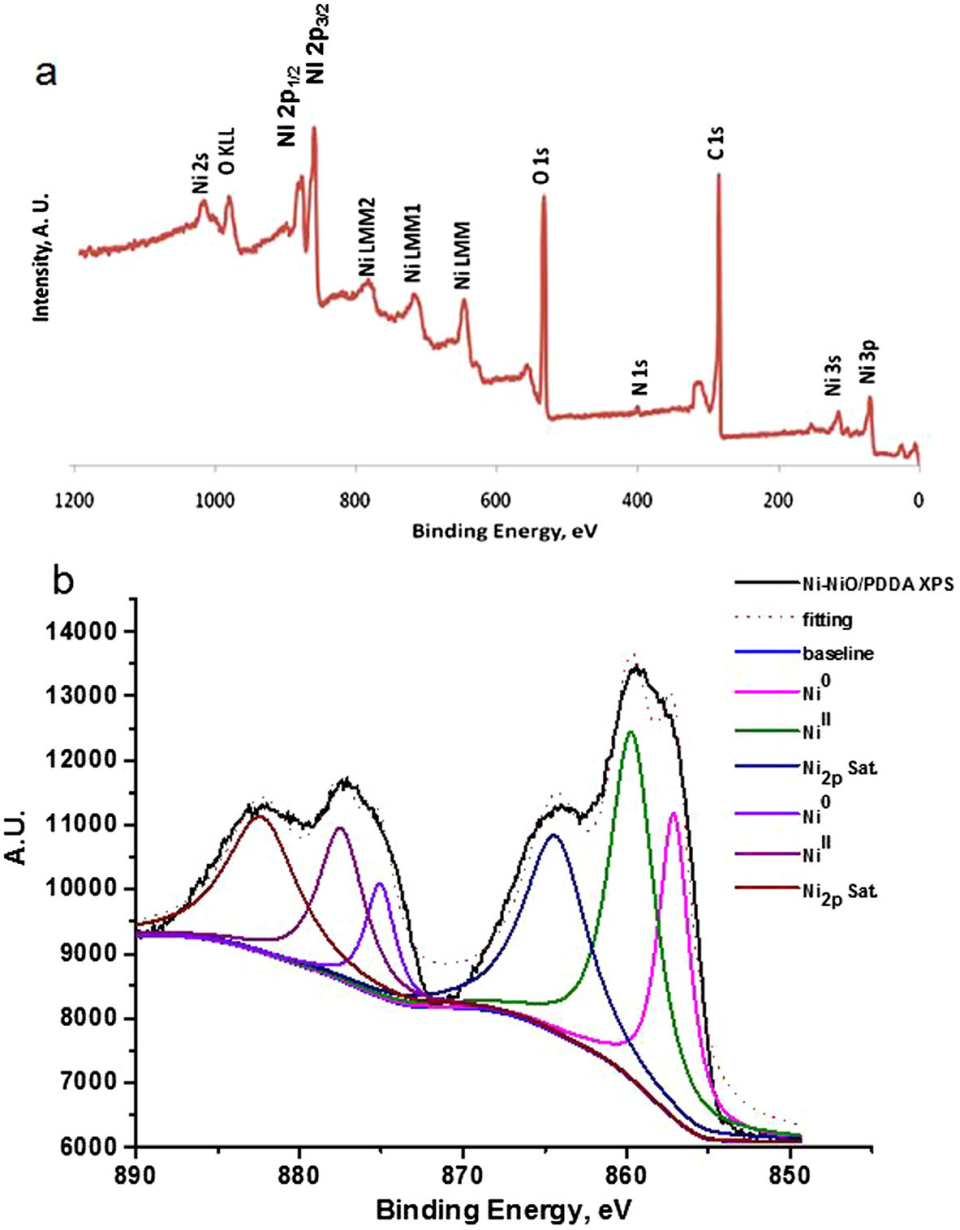
**ESCA/XPS spectrum of (a) survey scan and (b) Ni**_**2p **_**in the Ni-NiO/PDDA-G nanohybrids.**

PDDA was used to modify the surface of graphene, and then the Ni-NiO nanoparticles could be embedded on the PDDA-G surface. The change of functional groups in the Ni-NiO/PDDA-G would be evaluated by ESCA/XPS in Figure 4a. The C_1s_ binding energy of the C-C sp^2^ (284.6 eV, 72.4%) and that of epoxy group (286.7 eV, 27.6%) are shown, respectively. The binding energy of O_1s_ was fitted as 531.2 eV (C-O-Ni, 18.9%), 532.1 eV (C = O/O-Ni, 26.4%), 533.5 eV (C-OH/C-O-C, 30.0%), and 535.0 eV (COOH, 24.7), respectively. The N_1s_ spectrum was fitted as 399.4 eV (binding PDDA, 54.4%) and 400.6 eV (free PDDA, 45.5%). Furthermore, the curve fitting of Ni_2p_ spectrum results in Figure 4b shows Ni^0^ (the binding energies for 857.1 and 875.0 eV) and Ni^II^ (the binding energies for 859.7 and 877.5 eV) with the fitting ratio of 41.7% and 52.3%, respectively.

**Figure 5:**
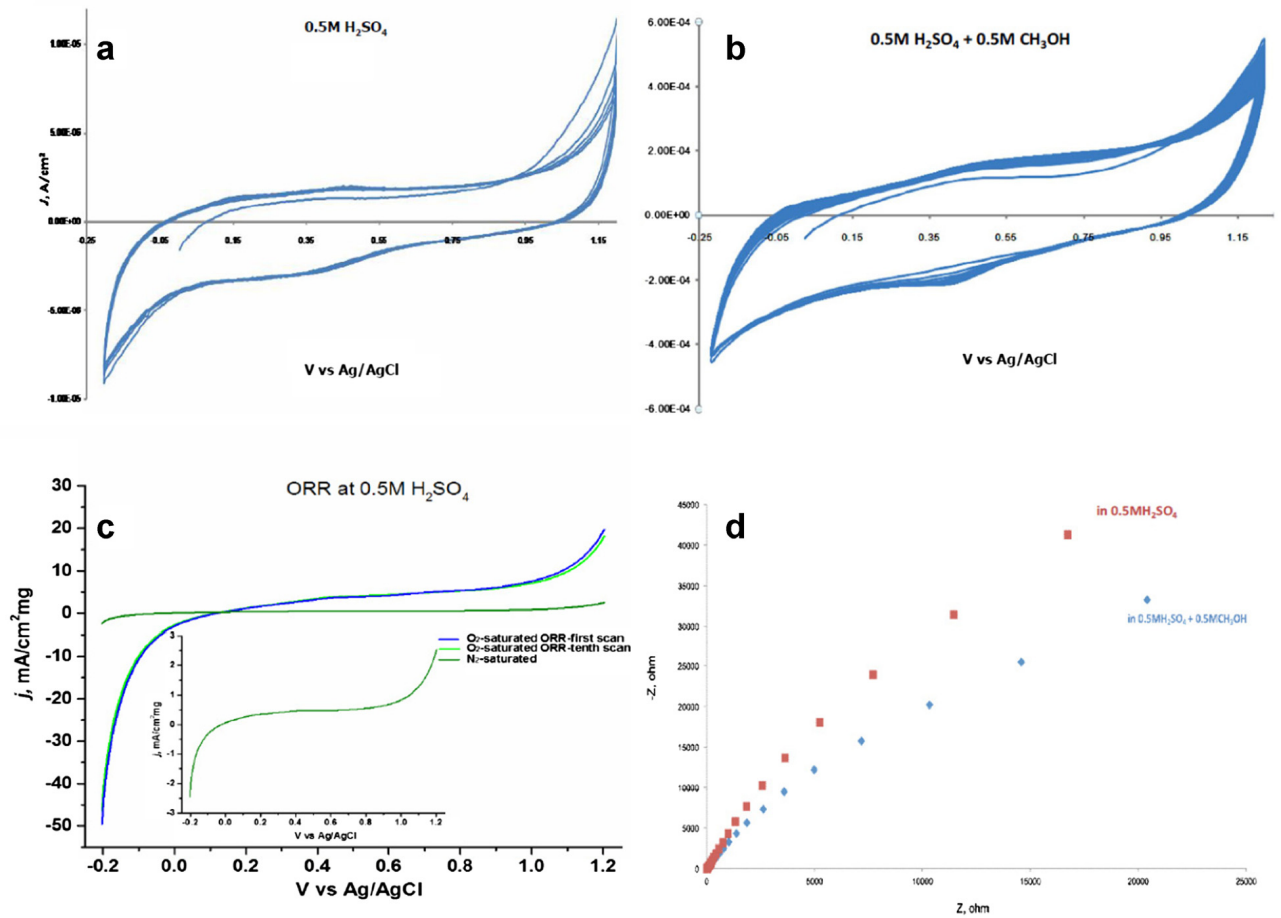
**The electrochemical studies of Ni-NiO/PDDA-G nanohybrids. (a)** CV in the 0.5 M aqueous H_2_SO_4_, **(b)** CV in the 0.5 M aqueous H_2_SO_4_ + 0.5 M CH_3_OH, **(c)** ORR test in the O_2_-saturated 0.5 M aqueous H_2_SO_4_, and **(d)** the EIS spectrum at −0.3 V.

The electrochemical investigation of Ni-NiO/PDDA-G was applied in the 0.5 M aqueous H_2_SO_4_ (shown in Figure 5a), 0.5 M aqueous H_2_SO_4_ + 0.5 M CH_3_OH (shown in Figure 5b), and the O_2_-saturated 0.5 M aqueous H_2_SO_4_ (shown in Figure 5c). Figure 5c shows no significant difference, as evidenced by the blue line denoting the O_2_-saturated ORR first scan and the green line denoting the tenth scan. The inset in Figure 5c is the ORR test in the N_2_-saturated 0.5 M aqueous H_2_SO_4_. The O_2_-saturated ORR test current density at the −0.2 to 0.2 V vs. Ag/AgCl is about 25 times than that of the N_2_-saturated ORR test of Ni-NiO/PDDA-G. Furthermore, the O_2_-saturated ORR test current density at the 1.0 to 1.2 V vs. Ag/AgCl is about 5 times than that of the N_2_-saturated ORR test of Ni-NiO/PDDA-G. The electrochemical impedance spectroscopy result for testing the 0.5 M aqueous H_2_SO_4_ and 0.5 M aqueous H_2_SO_4_ + 0.5 M CH_3_OH is shown in Figure 5d. The semicircle curve of Ni-NiO/PDDA-G in the 0.5 M aqueous H_2_SO_4_ is higher than that in the 0.5 M aqueous H_2_SO_4_ + 0.5 M CH_3_OH, showing the higher chemical reaction ability. Thus, the Ni-NiO/PDDA-G is more suitable for ORR than for the methanol oxygen reaction.

## Conclusions

We have successfully synthesized the Ni-NiO/PDDA-G nanohybrids, and the size of Ni-NiO nanoparticles was about 2 to 5 nm. The morphologies and chemical composition of Ni-NiO/PDDA-G were evaluated by TGA, XRD, TEM, and ESCA/XPS. The results show the phase of the Ni-NiO/PDDA-G, and the loading content of Ni-NiO is about 35 wt%. The CV and EIS results of Ni-NiO/PDDA-G in 0.5 M aqueous H_2_SO_4_ are better than those in 0.5 M aqueous H_2_SO_4_ + 0.5 M CH_3_OH. Therefore, Ni-NiO/PDDA-G in 0.5 M aqueous H_2_SO_4_ is more suitable as ORR electrocatalyst and could be a candidate of for cathode electrocatalyst of fuel cells.

## Competing interests

The authors declare that they have no competing interests.

## Authors' contributions

TYY, LYH, and TYL conceived and designed the experiments. PTC, LYH, TYC, and KSW performed the experiments. TYY, LYH, TYC, CYC, and KSW contributed ideas and material analyses. TYY, TYL, and LKL wrote the manuscript. This work was performed under the supervision of LKL. All authors read and approved the final manuscript.

## Authors’ information

TYY is an assistant engineer at the Institute of Nuclear Energy Research. LYH is a postdoctoral fellow at National Taiwan University of Science and Technology. PTC is a postdoctoral fellow at National Taiwan University. CYC is an associate professor at National Taiwan University. TYC and KSW are undergraduate students at Ming Chi University of Technology. TYL holds an assistant professor position at Ming Chi University of Technology. LKL is a research fellow at Academia Sinica and an adjunct professor at National Taiwan University.
